# Social Stigmatization of Drug Abusers in a Developing Country: A Cross-Sectional Study

**DOI:** 10.7759/cureus.10661

**Published:** 2020-09-26

**Authors:** Farhan Khalid, Ali Jaan, Mian Muhammad Salman Aslam, Zahoor Ahmed, Aniqa Raheem, Zubair H Bodla, Abdul Basit, Bilal Hussain, Aqsa Iftikhar, Muhammad Tayyeb, Awais Khalid, Usama Rehman

**Affiliations:** 1 Internal Medicine, King Edward Medical University, Mayo Hospital, Lahore, PAK; 2 Internal Medicine, Shalamar Medical and Dental College, Lahore, PAK; 3 Child Psychiatry, King Edward Medical University, Mayo Hospital, Lahore, PAK; 4 Psychiatry, Allama Iqbal Medical College, Lahore, PAK; 5 Anaesthesia, King Edward Medical University, Mayo Hospital, Lahore, PAK

**Keywords:** drug abusers, drug addiction, social stigma, addiction psychiatry

## Abstract

Introduction: Stigma is a label that differentiates a person from others and associates them with unenviable attributes. There are various forms of stigma: enacted stigma, perceived stigma, and self-stigma manifesting as stereotyping and harboring negative thoughts about the stigmatized group. Stigmatization of the drug abuser leads to effects such as low self-esteem, depression, and personality changes in the stigmatized.

Objectives: The purpose of the study is to know the impact of stigma on patients receiving substance abuse treatment in the Psychiatry Department, Mayo Hospital Lahore, Pakistan.

Design: Cross-sectional study.

Place: Psychiatry Department, Mayo Hospital, Lahore, Pakistan

Study Period: Six months (February 22, 2020, to July 18, 2020).

Subjects and Methods: A population-based cross-sectional study was conducted in a tertiary care hospital. A total of 100 patients were recruited in the study. The selection was made on laid down criterion after taking due consent. Interviews were conducted through a pretested questionnaire. Data were collected, compiled, and analyzed through SPSS version 20 (IBM Corp., Armonk, USA), and relevant frequency tables were drawn.

Results: On analyzing the data, various forms of stigma were observed: enacted stigma (81% as considered less capable), perceived stigma (99% as having difficulties in the job seeking and relationships), and self-stigma (94% in having devaluation thoughts). Self-esteem was maintained (73% were content). Social support was present (76% from family). Moderate depression was seen in 17% of participants. Over 83% of our study population is aged 21 to 40 years, 15% between ages 41 and 60 years, and only 2% ranging between ages 1 and 20. A total of 80% of the population belonged to a low socioeconomic status, and 55% of participants abused opium, heroin, or brown sugar, followed by white crystal use in 37% of the study population. The majority reported the drug abuse duration of 1-5 years (70%).

Conclusion: Stigma in its various forms affects the drug abuser undergoing treatment. It results in low self-esteem and mild depression. Individuals from the broader socioeconomic range can be added in future studies, and a larger population can be studied by collecting data from other tertiary care hospitals and mental healthcare facilities. They can be assessed for factors contributing to their addiction and the challenges they had to go through to get the help they needed.

## Introduction

Merriam Webster dictionary defines stigma as a mark of disgrace and shame [1]. Stigmatization is an interactive social process with people's attitudes affecting a person with a particular trait or habit. A drug abuser is an example of stigmatization. Drug abuse has dire social and moral repercussions, even when the person is undergoing treatment due to its stigma. The stigma becomes crucial because of the negative effect it has on the drug abuser in ways more than one, such as difficulties in job employment, interpersonal rejection, devaluing thoughts about oneself, seclusion, or avoidance of intimate contacts.

Research conducted in New York on the consequences of stigma showed that 6% of participants were denied medical treatment, 16% were denied housing, and 24% were paid lower wages because of a history of drug abuse [2]. A study showed that 31% of the patients were stigmatized by their family and friends, 18.9% by coworkers, 28.4% by healthcare workers, and 21.6% by others [3]. A research conducted in Nevada showed that drug abusers experienced unfair treatment (60%) and family rejection (45%) [4]. In a similar study, perceived stigma was prevalent in 85%, avoidance and seclusion in 74%, and social support by friends and family in 45% [5]. A study done in America on the effect of stigma on alcoholics showed that stigma was highest in men with low socioeconomic status [6]. A study conducted in Puerto Rico showed that 18.7% of health professionals believed that drug addicts are disagreeable patients [7]. In research conducted in the United States of America in mentally ill patients, participants reported discrimination (74%) and unreliability (69%) [8]. A study conducted in Chennai, India, showed that 67% of the people felt that they needed to hide their drug use, and 70% felt ashamed of using drugs [9]. In a similar study, 30% of participants felt negative social support, and 41.4% with fear of treatment [10]. Another research reported that increased exposure to illicit drugs was associated with an increased risk of lifetime use of those drugs between 35% and 49% [11,12]. A study done in New York proposed that awareness among the masses could lead to better interaction with the stigmatized people [13]. Another study showed that about 70% of the respondents had feelings of self-stigma and worthlessness [14]. Some studies have been reported on the prevalence of drug abuse in Pakistan. It was found out that the most commonly abused drugs included tobacco, cannabis, cocaine, opium, and heroin; 80% of the drug abusers were males, while 20% were females. The most common behavior associated with the stigmatized people was the use of intravenous needles [13,15-20].

This paper aimed to determine whether there were any enduring effects of stigma, even when treatment effectively reduces substance abuse. Our study outlined the factors which play a vital role in the stigmatization of drug abusers that will help shift the approach of treatment providers, health professionals, and family members towards the drug abuser; that will also help spread knowledge and awareness among the masses regarding this social issue. Moreover, many productive studies can be based on this research's results to ascertain the factors involved in drug abusers' stigmatization.

## Materials and methods

A cross-sectional study was conducted to determine the impact of disgrace on patients receiving substance abuse treatment in the Psychiatry Department, Mayo Hospital, Lahore, Pakistan from February 22, 2020, to July 18, 2020. A total of 100 subjects were included using a simple random sampling technique. Inclusion criteria included male drug abusers aged 18 to 70 years being treated for substance abuse. Exclusion criteria included females, uncooperative patients, and tobacco and caffeine abusers. Written consent was obtained from all the selected subjects. Data were collected by interviews using a pretested questionnaire containing questions representing various variables while keeping all the ethical and social considerations. The variables were defined as: stigma is the interdependence of five attributes, i.e., labeling, stereotyping, isolation, status loss, and bigotry. Perceived stigma is the belief that specific characteristics will always be found in association with a particular group of individuals [21]. Self-stigma encompasses the negative feelings, the person being stigmatized feels about himself, leading to low self-esteem and a lack of confidence, resulting in moral, physical, and mental deterioration [22]. Enacted stigma is the kind of stigma that the person receiving faces in his everyday life, like interpersonal rejection, seclusion, and social discrimination [22].

## Results

According to our findings, 83% of the study population is between ages 21 and 40 years, 15% between ages 41 and 60 years, and only 2% ranging between ages 1 and 20 years. A total of 80% belong to low socioeconomic status with monthly income below 150 $, and 20% belong to moderate socioeconomic status with monthly income above 150 $. Heroin, opium, and brown sugar are among the most commonly abused substances comprising 55% of our study population. A total of 37% reported the abuse of white crystal and 6% marijuana. The majority reported the drug abuse duration of 1-5 years (70%) (Table 1).

**Table 1:** Demographics

Variables	Frequency	Percentages
Age groups		
1-20	2	2
21-40	83	83
41-60	15	15
Total	100	100
Socioeconomic status		
Lower (below 150$./month)	80	80
Moderate (above 150$/month)	20	20
Total	100	100
Drugs abused		
White crystal	37	37
Heroin, opium, brown sugar	55	55
Marijuana	6	6
Total	100	100
Years abused		
1-5 years	70	70
6-10 years	20	20
11-15 years	5	5
16-20 years	3	3
21-25 years	1	1
26-30 years	1	1
Total	100	100

A total of 80% reported that they were considered less capable, 71% received bad comments, 52% were not treated nicely, 40% concealed their treatment history, 41% were avoided by people in social situations, and 48% were made to feel low when people came to know about their treatment history (Table 2).

**Table 2:** Enacted Stigma

S. No	Questions	Yes (n)	No (n)
1	Receiving bad comments	71	29
2	Being treated nicely	48	52
3	Concealing treatment	40	60
4	Less capable	80	20
5	Avoided by people	41	59
6	Made to feel low	48	52

A total of 99% reported that people were highly unlikely to befriend or trust them. All the patients thought that they were unlikely to get hired as teachers or caretakers of children. A total of 97% reported that most people think low of a substance abuser, 90% felt that an average individual was preferred over a person treated for substance abuse by society in general, and 73% felt that most people would not be willing to marry a person with a history of substance abuse (Table 3). A total of 76% hide substance abuse from their family, 94% thought they had destroyed their lives, and 77% thought that most people would not consider them a drug addict if they stopped taking drugs. A total of 70% reported being insulted and 67% reported labeled by others. A total of 57% responded that stress was not the reason for their substance abuse. Over 72% felt that people refused to give them time or money, and 79% felt people thought low of their character (Table 4).

**Table 3:** Perceived Stigma

S. No	Questions	Yes (n)	No (n)
1	Befriending	1	99
2	Trust	1	99
3	Hire as a school teacher	0	100
4	Hire as caretaker of children	0	100
5	Think low	97	3
6	Average individual preferred over a substance abuser	90	10
7	Marrying a substance abuser	27	73

**Table 4:** Self Stigma

S. No	Questions	Yes (n)	No (n)
1	Hiding	76	24
2	Destroyed life	94	6
3	Considered addict after treatment	23	77
4	Insulted	70	30
5	Labeling	67	33
6	Refused time and money	72	28
7	Stress	43	57
8	Low character	79	21
9	Trustworthiness	13	87

A total of 87% felt they were trustworthy, 73% were content, and 64% thought that they were as important as others. Only 25% felt like a loser, and 93% wished they had more faith in themselves. A total of 96% were sure they had good qualities (Table 5). Over 76% got family support under all circumstances, 73% got emotional support from family, and 66% shared their family problems. A total of 45% got peer support, and only 33% talked about their difficulties with friends (Table 6). A total of 42% of the participants were not depressed, 41% were mildly depressed, and 17% showed moderate depression (Table 7).

**Table 5:** Self Esteem

S. No	Questions	Yes (n)	No (n)
1	Contentment	73	27
2	Loser	25	75
3	Important as the rest	64	36
4	Faith in oneself	93	7
5	Good qualities	96	4

**Table 6:** Social Support

S. No	Questions	Yes (n)	No (n)
1	Family support	76	24
2	Emotional support	73	27
3	Share problems	66	34
4	Peer support	45	55
5	Support by friends	33	67

**Table 7:** Depression

Depression	Frequency	Percentage
Normal range (25-49)	42	42
Mildly depressed (50-59)	41	41
Moderately depressed (60-69)	17	17
Total	100	100

## Discussion

We set out to determine the impact of stigmatization on substance abusers in our study. Our hypothesis has turned out partially successful, as 58% of the participants were reported mild to moderately depress due to the stigmatization they experienced. That could also be attributed to the low socioeconomic status (80%) to which many of our participants belong, and socioeconomic status could very well be a determining factor in the participants' overall mental health. In another study, the level of stigma perceived by illicit drug users has been shown to persist even when drug use is reduced or ended and remains strongly associated with mental health symptoms.

The participants reported almost similar positive responses to perceived stigma, with substance abuse being a factor in others not befriending those (99%), trusting them (99%), or hiring them for responsibility positions as school teachers and caretakers for children (100%) and even marrying them (73%). While the participants did report experiencing enacted stigma and self-stigma, most of them did not conceal the fact that they had undergone treatment for substance abuse (60%), even though initially hiding their substance abuse from their families before treatment (76%). They also stated that they were not avoided by people when they left treatment (59%), and most were hopeful that this negative perception would disappear once their treatment was completed (77%).

Enacted stigmatization, perceived stigmatization, and self-stigmatization impact the mental well-being of these substance abusers. The participants reported receiving social support (76%), and emotional support (73%) coming from their families while being shunned by peers in general (55%), and majority reporting good self-esteem, associating good qualities with themselves (96%), and considering themselves trustworthy (87%) despite the contrary perceived opinion. Substance abusers continue to face challenges as illicit drug users are seen as weak, immoral, and risk society. Our results are congruent with a previous study conducted on the effect of stigma on substance abusers, which showed participants subjected to enacted stigma (60%), rejection by friends (38%), and rejection by family (45%). As put forward by previous research, experiences of discrimination in drug abusers can range from considerable exclusions to put-downs and slights [3]. In the present study, most participants reported experiencing high levels of self-stigma. However, only 43% attributed their drug abuse to stress indicating that a high proportion (57%) indulged in this habit for other reasons. A total of 52% were subjected to enacted stigma in the form of being mistreated. Rejection by friends was 55%, and rejection by family, only 26%, indicating a social support system that comes from living together as a family. A majority (79%) experienced negative and devaluation thoughts about themselves. Another study done in a Methadone treatment site found out that friends and family-related rejection were about 69% [3]. The slight difference in the results is due to dissimilarities in the geographical area, socio-cultural background, and family values.

Most of the participants in our study were subjected to an alarmingly high level of perceived stigma manifesting as being discriminated against in friendships (99%) and in seeking employment (100%) and not being considered trustworthy (99%). A study was done in America to study how stigma influences mentally ill patients, the values of perceived stigma for the above variables were lower in comparison (66%, 81%, and 69%, respectively) [8]. This suggests that drug abuse is more negatively stereotyped and seen as a taboo in our country than mental illness, and for obvious reasons.

A total of 71% of the participants experienced enacted stigma in being labeled as drug addicts, and 72% encountered perceived stigma in being refused time and money. In a study conducted in the North Eastern United States, similar results were obtained, with 41.9% participants facing stereotyping and 50.5% being refused time and money [3]. A total of 40% of the participants felt fear, 76% felt self-stigma, and about 45% had social support. In contrast, a study done in 2006 found participants experiencing consternation (7.4%), self-stigma (4.5%), and social support (8.3%) [10]. Our results show that 41% of participants felt that most people think low of a substance abuser, and the majority of the participants (99%) responded that most employers would not hire them. Likewise, 73% reported that they would not be ideal candidates for marriage. In the research conducted on stigma and its consequences in America, participants agreed that they would be looked down upon (65%), will not be hired by employers (72%), and will not get married (62%) [2]. These findings are almost consistent.

Many participants of research conducted in India reported feelings of enactment and self-stigma because of their substance abuse. About 57% of the participants felt that people avoided them and did not want to be around them, signifying our results where 41% of the participants felt that most of the people kept away from them [9]. Almost 41% of the participants reported mild depression, while 17% were moderate to severely depressed. However, the majority (42%) did not experience any sign of depression, justifying that most of the patients recovering from substance abuse were hopeful about their future and content with themselves. No previous study has reported measuring depression in substance abusers experiencing stigma.

This study primarily dealt with these individuals' subjective experiences regarding their social interaction, and most of them belonged to low socioeconomic status. More studies can be done in the future, including individuals from a broader range of socioeconomic groups. Our study population only includes individuals being treated for substance abuse in a single tertiary care hospital. Our study included 100 patients only, and a larger population by including more participants from other tertiary care hospitals and mental healthcare facilities can also be done to remove bias. It was a single-center study that could result in further bias. We can also assess factors related to addiction-like family history, peer pressure, history of psychiatric illness, and how these drugs' availability contributed to their substance abuse. The stigma associated with addiction and its treatment is one of the major factors that stop people from getting the help that they need. We can also assess the difficulties these patients faced in the pursuit of proper treatment. Public awareness campaigns at a larger scale regarding factors that contribute to addiction can help change our perspective as a society, and we can work for their psychological and socioeconomic well-being. As our study design is a prevalence study, it only establishes an association, not the causation. Prevalence studies can be repeated to see the trend over time. Hypotheses can also be drawn from these cross-sectional studies for more complex studies like cohort studies, which can help establish causation.

## Conclusions

Stigma in its various forms affects the drug abusers undergoing treatment. It results in relatively low self-esteem and varying degrees of depression. However, the stigma associated with drug abuse decreases to some extent after getting treatment. The majority of the individuals in our study population associate good qualities to themselves and consider themselves trustworthy. This shows how access to proper treatment can help them deal with addiction and becoming productive members of our society. Social support by family and friends is attributed to an overall decreased experience of stigma. It is essential to bring awareness on a larger scale about risk factors leading to stigma. It is unfair to put the entire responsibility on them without addressing the genetic and environmental components associated with addiction. Such steps are crucial for the social, psychological, and economic well-being of individuals dealing with addiction.

## References

[1] (2020). Stigma. http://www.merriam-webster.com/dictionary/stigma.

[2] Link BG, Struening EL, Rahav M, Phelan JC, Nuttbrock L (1997). On stigma and its consequences: evidence from a longitudinal study of men with dual diagnoses of mental illness and substance abuse. J Health Soc Behav.

[3] Earnshaw V, Smith L, Copenhaver M (2013). Drug addiction stigma in the context of methadone maintenance therapy: an investigation into understudied sources of stigma. Int J Ment Health Addict.

[4] Link BG, Yang LH, Phelan JC, Collins PY (2004). Measuring mental illness stigma. Schizophr Bull.

[5] Ahern J, Stuber J, Galea S (2007). Stigma, discrimination and the health of illicit drug users. Drug Alcohol Depend.

[6] Keyes KM, Hatzenbuehler ML, McLaughlin KA, Link B, Olfson M, Grant BF, Hasin D (2010). Stigma and treatment for alcohol disorders in the United States. Am J Epidemiol.

[7] Varas-Díaz N, Santiago-Negrón S, Neilands TB, Cintrón-Bou F, Malavé-Rivera S (2010). Stigmatization of illicit drug use among Puerto Rican health professionals in training. P R Health Sci J.

[8] Link BG, Struening EL, Neese-Todd S, Asmussen S, Phelan JC (2016). Stigma as a barrier to recovery: The consequences of stigma for the self-esteem of people with mental illnesses. Psychiatr Serv.

[9] Latkin C, Srikrishnan AK, Yang C (2010). The relationship between drug use stigma and HIV injection risk behaviors among injection drug users in Chennai, India. Drug Alcohol Depend.

[10] Rapp RC, Xu J, Carr CA, Lane DT, Wang J, Carlson R (2006). Treatment barriers identified by substance abusers assessed at a centralized intake unit. J Subst Abuse Treat.

[11] Palamar JJ, Halkitis PN, Kiang MV (2013). Perceived public stigma and stigmatization in explaining lifetime illicit drug use among emerging adults. Addict Res Theory.

[12] Palamer JJ, Kiang MV, Halkitis PN (2012). Predictors of Stigmatization towards use of various illicit drugs among emerging adults. J Psychoactive Drugs.

[13] Link BG, Phelan JC (2001). Conceptualizing stigma. Annu Rev Sociol.

[14] Luoma JB, Nobles RH, Drake CE, Hayes SC, O'Hair A, Fletcher L, Kohlenberg BS (2013). Self-stigma in substance abuse: development of a new measure. J Psychopathol Behav Assess.

[15] Afzal S, Salman F, Ashraf S (2013). The determinants of smoking in females. Ann KEMU.

[16] Afzal S, Salman F, Khan A (2013). The determinants of shisha smoking in medical students. Ann KEMU.

[17] Alam SE (1998). Prevalence and pattern of smoking in Pakistan. J Pak Med Assoc.

[18] Ali S, Ara N, Ali A, Ali B, Kadir MM (2008). Knowledge and practices regarding cigarette smoking among adult women in a rural district of Sindh, Pakistan. J Pak Med Assoc.

[19] Rozi S, Akhtar S, Ali S, Khan J (2005). Prevalence and factors associated with current smoking among high school adolescents in Karachi, Pakistan. Southeast Asian J Trop Med Public Health.

[20] Altaf A, Shah SA, Zaidi NA, Memon A, Rehman N, Wray N (2007). High risk behaviors of injection drug users registered with harm reduction program in Karachi, Pakistan. Harm Reduct J.

[21] Luoma JB, Twohig MP, Waltz T, Hayes SC, Roget N, Padilla M, Fisher G (2007). An investigation of stigma in individuals receiving treatment for substance abuse. Addict Behav.

[22] Link BG, Cullen F, Struening E, Shrout P, Dohrenwend B (1989). A modified labeling theory approach to mental disorders. Am Sociol Rev.

